# Commentary: The ‘Musilanguage’ Model of Language Evolution

**DOI:** 10.3389/fpsyg.2018.00075

**Published:** 2018-02-26

**Authors:** Aleksey Nikolsky

**Affiliations:** Braavo Enterprises, Los Angeles, CA, United States

**Keywords:** texture, heterophony, polyphony, homophony, musilanguage, asynchrony, isophony, meter

The model of musilanguage (Brown, 2000, 2017) requires a new musicological term to refer to its texture. Like choral singing, and unlike speech, musilanguage is based on simultaneous vocalization of multiple participants who reproduce the same signal (call) at random time intervals and pitch levels, akin to a wolf “chorus.^1^” Voiced utterances produce multiple pitches, generating a “jumbled” texture, similar to polyphony and heterophony, but not fully qualifying as such.

The term “heterophony” was introduced by Stumpf (1897) to refer to the fusion of sounds whose components retained their singular identity. Stumpf discovered this word in Plato's *Laws* (Plato, 2013, p. 203), where it referred to pitch and rhythm discrepancy between the vocals and the lyre performing the same tune. Four years later, Stumpf (1901) reused this term to describe Thai music. He characterized *heterophony* as the looping of simultaneous melodic paraphrases [Umspielen], where parts generally followed the same melodic contour while differing in detail, so that minute discrepancies would meet again in unison.

Adler (1908) conceptualized heterophony as a style alternative to homophony and polyphony, applicable across Siamese, Japanese, Javanese, and Russian musics. He specifically found Russian heterophony to present a paradigm of heterophonic arrangement, designed to make melodic repetition less monotonous and more idiosyncratic for each singer's voice.

The Russian Musical Encyclopedia defines heterophony as a multi-part music generated by the collective performance of the same melody, where parts contain deviations from the principal melodic formula (Mueller, 1973). Such organization is regarded as a general textural type of ornamental, harmonic, and/or polyphonic variation that can complicate classification. Indeed, already in 1911, Stumpf criticized Adler for misapplication of the term (Stumpf and David, 2012). The keynote of heterophony is an ongoing melodic repetition with numerous intermittent variations, which seems to apply to musilanguage chorusing (Brown, 2007). However, such chorusing contains no synchronization, whereas heterophony implies prevalent synchronization of parts.

Swan (1943) defined heterophony as “a *principal melody* improvised *simultaneously* by several singers, *retaining its main outline in each voice*, yet showing enough independence to result *in places* in 2- and 3- and even 4-part harmony^2^.” The Grove Dictionary (Cooke, 2001), following Swan's definition, emphasizes a collective synchronized execution. Although the notation example provided in the Grove article shows a consistent misalignment of 5 parts (Knudsen, 1968), such heterophony is rare and does not sound “jumbled^3^.” Its asynchronicities remain minimal (<half-a-beat). Longer asynchronies (≥beat) generate polyphonic imitations, where the same melody becomes deliberately distributed between multiple parts to produce juxtapositions at certain temporal intervals^4^.

*Polyphony* is “a style of simultaneously combining number of parts, each forming an individual melody and harmonizing with each other” (Oxford Dictionary). Despite its association with Western art-music, polyphony penetrated Western popular (Bukofzer, 1940) and traditional music (Ahmedaja, 2011), prompting research of non-European polyphony (Arom, 2004; Jordania, 2006). Many ethnomusicologists prefer alternative terms (diaphony, disphony), while others treat “polyphony” as an umbrella term for any multi-part music, setting terminological confusion (Cooke, 2001). Current consensus defines polyphony as “a mode of expression based on simultaneous combination of *separate parts*, perceived and produced *intentionally* in their *mutual differentiation*, in a given *formal order*” (Agamennone, 1996)^5^.

Polyphonic and heterophonic textures differ in orderliness: heterophonic parts are inadvertent, unlike polyphonic parts (Tallmadge, 1984). Polyphony induces individualization of parts by means of sharpening their functional contrast in texture. Hence, synchronization is even more important for polyphony—parts must align in pitch and time throughout the entirety of music. This makes polyphonic performance metrically stricter than heterophonic performance.

Even stricter is synchronization in another “classic” texture—*homophony*—“music in which all melodic parts move together at more or less the same pace” (Hyer, 2001). Contrary to common belief, homophony is *not* bound to European music alone (Nikolsky, 2016). Its reliance on chords and harmonic intervals demands high concision in tones' onsets: in the order of under 100 msec (Huron, 2001), typically, 30–50 msec (Rasch, 1988).

All “classic” textures rely on *harmonic, metric, and thematic integrity of parts*. Performers attune their performance to the pitch of their partners, the manifestation of beat in their rhythms, and the distribution of musical material across parts—what musicologists call “thematic material” and consider an expressive point of a musical work by which it can be remembered (Drabkin, 2001). In this semiotic sense (Réti, 1951), the notion of *thematicity* is applicable to folk and non-Western music (Mazel, 1960). However, harmonicity, metricity, and thematicity are inadmissible for musilanguage. Even modifying “classic” terms (e.g., “jumbled heterophony”) would constitute a misnomer: musilanguage inherently lacks any form of arrangement of parts.

Since musilanguage occupies an evolutionary position between the “natural” animal vocalizations and the simplest human oral communication, it predates mode, scale, meter—and therefore, heterophony and polyphony. This situation calls for a new term—*isophony:* texture that uses brief calls, continuously reproduced by multiple performers with irrational deviations in timing and pitch, where each participant retains idiosyncrasy of the rhythmic, timbral, and directional attributes of the pitch contour—altogether producing a “jumbled” effect (Nikolsky, 2016, Appendix-5)^6^. Vocalization can be considered “isophonic” if it maintains a single call as a unit of texture, scalable shorter/longer and higher/lower through the continuum of duration and frequency for every participating part—consistently reproducing that call out-of-sync in relation to the moment of its onset or termination.

Isophony contrasts “classic” textures by its tendency to expose each participant's identity without enmeshing into the ensemble. Isophony involves the *assembly of individuals*, rather than a single entity (“choir”). Isophonic tones never meet in unison or in beat, and are devoid of any form of harmonization^7^. Isophony's only feature of tonal organization is the uniformity of the melodic and timbral characteristics of a call. The function of isophonic texture is to attract attention to each participant's expression of the same state of mind. The important features that distinguish isophony from heterophony, polyphony, and homophony are summarized in the Table 1.

**Table 1:** Feature comparison of 4 types of musical texture in a multi-part ensemble.

**Parts**	**Heterophony**	**Polyphony**	**Homophony**	**Isophony**
Numerosity	From 2 to a few dozen parts—basically, as many as there are participants. Each part is performed solo, but often merges with other parts to make a tutti unison.	From 2 to 3 (or 4) parts. In Western art-music, usually up to 8 (and rarely up to 40). A part can be performed by a soloist or a group. Each part is sustained throughout the entire music.	Usually from 2 to 5 parts—to make harmonic intervals or chords: chunks of tones, perceived as a single sonic entity. A part can be performed by a soloist or a group. It can cede and/or reappear later in music.	From 2 to an unlimited number of parts—as many as there are participants. Each part is performed solo, and it neither merges nor concords with other parts.
Thematicity	All parts carry out the same melody (tune)—yet, each with its own melodic details. The entire music sounds as numerous repetitions of the same theme.	Parts can use a single melody (imitational polyphony) or multiple melodies (contrasting polyphony), both of which sound continuously changing.	One part carries out the melody against the other parts that form a uniform accompaniment. The melody usually contains diverse thematic material, but can be repetitive.	All parts reproduce the same call (phrase) with variations in timing and pitch. The overall sound is more cluttered (less repetitive) than in heterophony.
Synchrony	Parts are synced at the initiation and termination points of the melodic phrases.	Parts are synced all the way from the beginning to the end of the music, in the order of a beat.	Parts are synced to make chords and harmonic intervals—with precision of 30–100 milliseconds.	Parts are not synced at all—each part freely proceeds at its own pace.
Pitch relation affordances	Each part can comprise any harmonic interval in relation to another part—but stays mostly in unison.	Each part is restricted to specific harmonic intervals in relation to another part—mostly non-unison.	Each part is restricted to specific harmonic intervals in relation to another part—mostly 5th, 3rd, 6th, and more seldom–4th.	Each part comprises any interval in relation to another part—tuned and untuned.
Time relation affordances	Parts stay metrically coordinated, while allowing each part to have its own rhythmic variation and expressive timing (e.g., rubato, ritenuto, fermata).	Parts stay metrically and rhythmically coordinated by means of the metric division system, leaving very limited margins for expressive timing per part.	Parts stay metrically and rhythmically coordinated, while letting melody and accompaniment each have their own autonomous rhythmic variation and expressive timing.	Parts stay metrically and rhythmically uncoordinated, each with its own variation and expressive timing.
Intentionality	The relationship between parts is not determined by the performers, but is rather, an emergent property of their attempt to perform the same melody.	The relationship between parts is governed by a complex set of melodic and harmonic rules that constrain the performers in their performance.	The relationship between the accompanying parts and the melodic part is governed by a simpler set of harmonic rules, binding the performers.	The relationship between parts is not consciously controlled by any of the participating performers, but rather emerges purely per chance.
Functionality	All parts are functionally identical in presenting the same melodic idea, with minute variations in pitch and rhythm. Each part is equal in status to other parts, where they all collaborate to continuously maintain the same expression throughout the music in a rather static way.	Imitative polyphony presents a single melodic idea expressed in succession of “call” and “response,” where “call” subdues “response.” Contrasting polyphony presents multiple melodies with diverse functions. All polyphonic parts compete for attention, promoting melodic development.	Melodic part presents the principal idea which is usually followed by secondary melodic ideas. Other parts can all carry the same accompanying function, or be specialized (e.g., bass, pedal, melodic figuration). Parts can switch their functions and regroup—thereby generating contrast, development, and recapitulation.	All parts contain the same call that is repeated with substantial changes in frequency and duration. Each part is equal in status to other parts in producing an independent expression of the same idea by each performer—in the manner of reaffirming this idea.

Conceptualization of isophony as a primordial texture that predated music, establishes the lineage in the morphological evolution of music, allowing comparative cross-examination of musical structures in multi-part music.

## Author contributions

The author confirms being the sole contributor of this work and approved it for publication.

### Conflict of interest statement

AN was employed by company, Braavo Enterprises as a technical and creative director. Braavo Enterprises specializes in developing content for educational and edutainment programs for children of 5–11 years of age. The author declares that the research was conducted in the absence of any commercial or financial relationships that could be construed as a potential conflict of interest.

## Footnotes

^1^The sample of a wolf pack howling can be heard at:http://chirb.it/kFLxIC. It is characterized by the reproduction of the same call at various speeds and pitch levels, without any synchronization of the onset and termination points of the calls.

^2^I have italicized those words in the definition that imply the general coordination in time between the performances of all the participants.

^3^An example of the Hebrides Psalm can be heard at: http://chirb.it/raeaOr. It presents a clear single melodic line that is “smudged” by consistent delays between different parts—presenting a rare style of melodic variation by means of a deliberate “reverberation” effect induced by the continuous sub-beat time lagging between parts.

^4^Well-known examples of musical texture in monothematic imitational polyphony in folk music are round-songs. In art-music, Bach's “Musical Offering” BWV 1079 contains excellent demonstration of polyphonic arrangement of a single melody in the so-called “riddle canons”—notated as a melody solo ought to be performed by a few musicians who enter one by one in specific time intervals.

^5^My italics emphasize points that distinguish polyphonic texture from heterophonic.

^6^This appendix contains an overview of the evolution of musical texture, following the model of “histories of fine art”—see Table 2 (p. 13–16) in it.

^7^An example of isophony is an Akia tribe song (courtesy of Anthony Seeger): http://chirb.it/ryANbJ. The leader coins the call (vocable “Tete”), and the tribe repeats it at various pitch levels and duration values, where each participant displays their specific social status through the relative duration of the same call (Seeger, 2004).
